# Genetics, Insurance and Professional Practice: Survey of the Australasian Clinical Genetics Workforce

**DOI:** 10.3389/fpubh.2018.00333

**Published:** 2018-11-23

**Authors:** Jane Tiller, Louise Keogh, Samantha Wake, Martin Delatycki, Margaret Otlowski, Paul Lacaze

**Affiliations:** ^1^Public Health Genomics, Department of Epidemiology and Preventive Medicine, School of Public Health and Preventive Medicine, Monash University, Melbourne, VIC, Australia; ^2^The University of Melbourne, Melbourne, VIC, Australia; ^3^Centre for Health Equity, Melbourne School of Population and Global Health, The University of Melbourne, Melbourne, VIC, Australia; ^4^Victorian Clinical Genetics Services, Parkville, VIC, Australia; ^5^Bruce Lefroy Centre, Murdoch Children's Research Institute, Melbourne, VIC, Australia; ^6^Royal Children's Hospital, Parkville, VIC, Australia; ^7^Faculty of Law, Centre for Law and Genetics, University of Tasmania, Hobart, TAS, Australia

**Keywords:** insurance, life insurance, genetics, genetic discrimination, genetic counselling, regulation, Australia

## Abstract

In Australia and New Zealand, by contrast with much of the developed world, insurance companies can use genetic test results to refuse cover or increase premiums for mutually-rated insurance products, including life, income protection and disability insurance. Genetics professionals regularly discuss insurance implications with clients and report the issue as a clinical challenge, yet no studies have examined clinical practices or opinions. This study surveyed genetic counsellors and clinical geneticists from Australia and New Zealand to (i) investigate variability in professional practice across the Australasian clinical genetic workforce relating to the insurance implications of genetic testing, and (ii) ascertain views regarding current regulation of the issue. There was considerable variability in training and clinical policies, especially around the communication of insurance implications. Almost half of participants reported receiving no training on the insurance implications of genetic testing, and almost 40% were unsure whether they could adequately advise clients. A number of deficits in professional knowledge and understanding of the issue were identified. Widespread concerns regarding regulation of this area were reported, with < 10% of Australian participants considering current Australian regulations as adequate to protect clients from genetic discrimination. The findings from this study highlight scope for greater education, consistency and professional training on the issue of genetics and insurance in Australasia, and strong agreement about the need for regulatory reform.

## Introduction

In Australia and New Zealand, insurance companies can use genetic test results to refuse cover, increase premiums or exclude aspects of cover for mutually-rated life insurance products, including life, income protection and total disability insurance. Genetic test results cannot be used for health insurance in Australia, which is community rated (1), but can be used in New Zealand for this purpose.

Many countries, including Canada, the UK and much of Europe, have banned or restricted the use of genetic information by life insurance companies (2, 3). In Australasia^1^ however, life insurance companies can require applicants to disclose any results of genetic testing known to the applicant. This includes genetic results from clinical testing as well as research and online, direct-to-consumer genetic tests (4). Insurers can then use that information, with other health and lifestyle information, in making underwriting decisions.

The use of genetic test results by life insurers is particularly relevant for individuals who are unaffected by disease and undergoing clinical predictive genetic testing (e.g., for neurogenetic conditions, such as Huntington disease or cancer predisposition, such as Lynch syndrome). Emerging research demonstrates that some at-risk individuals are deterred from having predictive genetic testing (5, 6) and choosing not to participate in genomic research (7) because of insurance fears.

Life insurers in Australia and New Zealand are currently self-regulated [managed by the peak industry body in each country, both named the Financial Services Council (FSC)], without government oversight (8). It can be argued that this creates uncertainty for consumers and genetics professionals regarding how insurers will use genetic information and raises numerous other concerns which have been discussed elsewhere (8). It is argued that the Australian FSC's recent policy changes are only likely to increase this uncertainty, as the new policy recommends insurers ask whether applicants are “considering” a genetic test. Given the applicant at this stage has no knowledge of genetic test information that the insurer does not have, there would appear to be no imbalance of information if consideration of a genetic test is not revealed to the insurer (8).

Clinical genetics professionals are in a unique position to inform clients about insurance implications of genetic testing before testing takes place (9). Guidance from the Human Genetics Society of Australasia (HGSA), the representative body for human genetics professionals in Australia and New Zealand, indicates genetics professionals should include a discussion of relevant insurance issues during consultations (10, 11). Two published Australian studies have shown that genetics professionals routinely discuss life insurance implications with clients during pre-test counselling sessions (12, 13). This takes time in sessions that cover a significant amount of information; however, to our knowledge there are no Australasian studies exploring professional practice in this area.

This study was designed to determine if variability exists in workplace trends, training policies and opinions related to the issue of genetic testing and insurance, and its current regulation.

## Methods

### Participants

Genetics professionals were recruited through the HGSA by email to members of the Australasian Society of Genetic Counsellors and the Australasian Association of Clinical Geneticists, the HGSA newsletter, and the 2017 HGSA Annual Scientific Meeting. Although the focus of the project was on Australian practice, the HGSA includes Australian and New Zealand practitioners and any interested participants were encouraged to participate. A screening question was used to include only genetics professionals who see clients considering genetic testing.

### Data collection and analysis

The study utilised an online survey (Appendix 1 in Supplementary Material), which was developed and refined through consultation with statistical and subject matter experts, including genetic counsellors, geneticists, and law and ethics experts. The survey aimed to measure (1) presence and adequacy of training and policies held by genetics services regarding communication of insurance issues with clients; (2) knowledge and practice of genetics professionals; and (3) attitudes regarding regulation of the area. The published literature was reviewed and relevant validated scales were considered, however, no scales were suitable for the topics in the survey.

The survey was open for data collection from 7 June 2017 until 18 August 2017. Data were collected and managed using REDCap (Research Electronic Data Capture) electronic data capture tools hosted at Murdoch Children's Research Institute (14). Online survey data were de-identified and exported for analysis using STATA 14 (StataCorp, Texas). No calculations related to power or statistical significance were performed for this exploratory study. Qualitative data were collected from selected participants through telephone interviews, but these data are not reported in this paper.

### Ethics committee approval

This study was completed in partial fulfilment of the requirements for the Master of Genetic Counselling, University of Melbourne, Victoria, Australia, and was supported by the Victorian Government's Operational Infrastructure Support Program. Approval for the project was granted by the Human Ethics Advisory Committee, Department of Paediatrics, University of Melbourne on 12 May 2017.

## Results

### Participant response

Eighty-seven genetics professionals participated in the online survey. The number of participants who completed each question (*n* value) is reported. The demographics of the survey participants are set out in Table 1.

**Table 1:** Participant demographics.

**Demographic**	**Category**	**Number of online survey participants (*n* = 87)**
Gender	Male	8 (9%)
	Female	79 (91%)
Profession	Medically trained genetics professionals	15 (17%)
	Genetic counsellors	72 (83%)
Years of experience	0–5 years	34 (39%)
	6–10 years	17 (20%)
	11–15 years	14 (16%)
	15–20 years	15 (17%)
	>20 years	7 (8%)
Appointments per fortnight	0–5	13 (15%)
	6–10	37 (42%)
	11–20	31 (36%)
	>20	6 (7%)
Location	Australian Capital Territory	1 (1%)
	New South Wales	23 (27%)
	New Zealand	6 (7%)
	Queensland	7 (8%)
	Tasmania	2 (2%)
	South Australia	6 (7%)
	Victoria	27 (31%)
	Western Australia	15 (17%)

**Figure 1:**
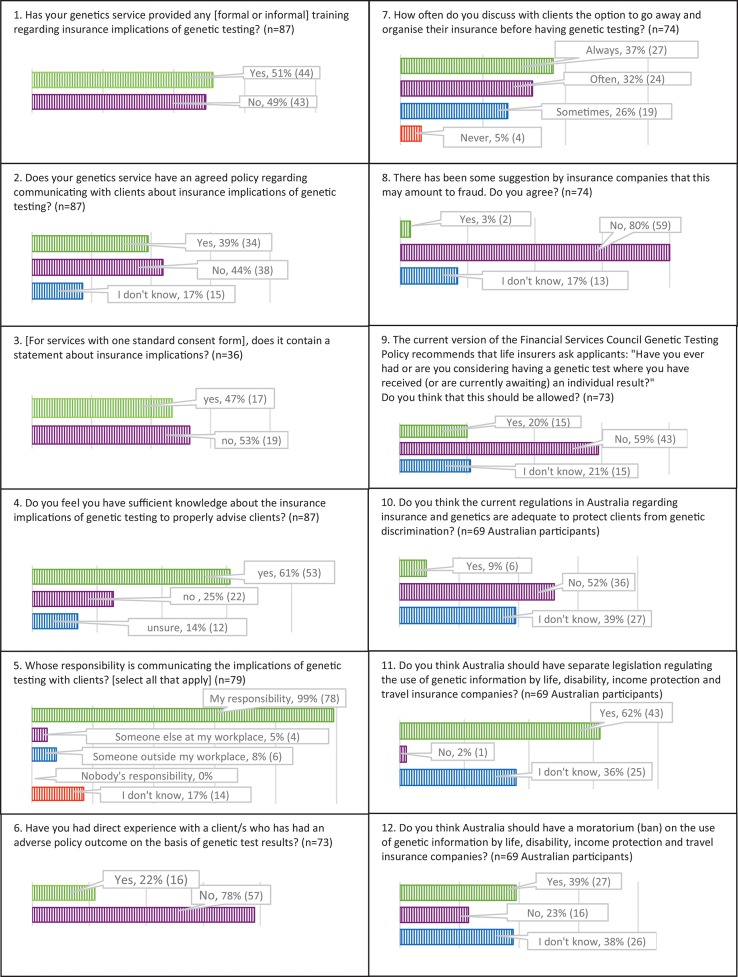
Results about training, policy, knowledge, professional practice, and views on regulation.

Figure 1 summarises results about training, policy, knowledge, professional practice and views on regulation presented below.

### Training, policy, knowledge

Forty-nine percent (*n* = 43/87) of participants reported the genetics service where they work had not provided training about the insurance implications of genetic testing (Figure 1, box 1), and 20% of participants who had received training (*n* = 9/44) felt this training was inadequate. Sixty-one percent (*n* = 53/87) of participants stated that either their genetics service did not have a policy (44%, *n* = 38/87) or they were unsure whether there was a policy (17%, *n* = 15/87) regarding communicating with clients about the insurance implications of genetic testing (Figure 1, box 2).

Forty-six percent of participants (*n* = 36/79) indicated their genetics service has one standard consent form for all types of genetic testing, and 53% (*n* = 19/36) of these do not include a statement about insurance implications (Figure 1, box 3).

Thirty-nine percent (*n* = 34/87) of participants did not have (25%, *n* = 22/87) or were unsure of having (14%, *n* = 12/87) sufficient knowledge about the insurance implications of genetic testing to properly advise clients (Figure 1, box 4). Of these, 71% (*n* = 24/34) had < 10 years' professional experience. Of participants with more than 10 years' experience, 27% (*n* = 10/36) did not have or were unsure of having sufficient knowledge. Fifteen percent of Australian participants (*n* = 11/74) believe that genetic information could be used for health insurance policies in Australia,^2^ which is incorrect (1). Ninety-three percent (*n* = 74/79) of participants stated it could be used for life insurance, 68% (*n* = 54/79) for disability insurance, 91% (*n* = 72/79) for income protection insurance and 42% (*n* = 33/79) for travel insurance, indicating variability in knowledge of current regulation. Travel insurance is a mutually rated insurance product,^3^ meaning providers can use genetic test results to assess risk, though their decisions, in theory, must have a reasonable basis. Three participants of 79 (4%) did not know which insurance policies genetic information could be used for.

Eighty-five percent (*n* = 68/80) of participants had read the Centre for Genetics Education (CGE)'s Fact Sheet 20 titled, “Life insurance products and genetic testing in Australia” (15). This document, to the authors' knowledge, is the most comprehensive resource currently available to professionals and the public on this issue. Eleven of the 12 participants who had not read the Fact Sheet were not made aware of its existence by their workplace.

### Professional practice

All participants stated that communicating information about insurance implications of predictive genetic testing in adults is very important (*n* = 60/79) or somewhat important (*n* = 19/79), and 99% (*n* = 78/79) consider that this communication is their responsibility (Figure 1, box 5).

Ninety-four percent (*n* = 74/79) of participants always discuss insurance implications with unaffected adults considering predictive genetic testing. Practice differed for other kinds of testing (diagnostic testing in children/adults, predictive testing in children, and prenatal testing). Participants were next most likely to discuss insurance implications in predictive testing in unaffected children (75% always discussed, *n* = 59/79) and least likely in prenatal testing (3% always discussed, *n* = 2/79).

### Use of genetic results by insurers and regulation

Twenty-two percent (*n* = 16/73) of participants have had direct experience with a client/s who had an adverse policy outcome on the basis of genetic test results (Figure 1, box 6). Ninety-five percent of participants (*n* = 70/74) sometimes (*n* = 19/74), often (*n* = 24/74) or always (*n* = 27/74) discuss with clients the option to go away and organise their insurance before having genetic testing (Figure 1, box 7). Only three percent of participants (*n* = 2/74) agreed with the suggestion that this practice may amount to fraud (Figure 1, box 8).

Fifty-nine percent (*n* = 43/73) of survey participants considered insurers should not be allowed to ask whether applicants are considering having a genetic test, while 21% (*n* = 15/43) thought it should be allowed. Twenty percent (*n* = 15/73) were unsure (Figure 1, box 9).

Of the Australian participants surveyed, 9% (*n* = 6/69) considered current Australian regulations adequate to protect clients from genetic discrimination. Fifty-two percent (*n* = 36/69) felt they were inadequate and 39% (*n* = 27/69) were unsure (Figure 1, box 10). When asked about types of regulation that could be implemented, 62% (*n*=43/69) considered Australia should have separate legislation regulating the use of genetic information by insurers. Two percent (*n* = 1/69) of participants answered no to this question, and 36% (*n* = 25/69) were unsure (Figure 1, box 11). Thirty-nine percent (*n* = 27/69) of participants considered Australia should ban the use of genetic information by insurers, while 23% (*n* = 16/69) did not agree with a ban and 38% (*n* = 26/69) were unsure (Figure 1, box 12).

## Discussion

Results of this study suggest many Australasian genetics professionals, while acknowledging it as a major issue in clinical practice, do not feel adequately equipped to advise clients regarding the insurance implications of genetic testing.

### Practice implications

Genetic professionals have a fundamental obligation to promote informed consent and ensure clients understand the implications of genetic testing (16). Where genetics professionals have either self-declared, or demonstrated through incorrect survey responses, a lack of knowledge, the implications are significant for their practice. Although the majority of professionals self-reporting inadequate knowledge in this area had < 10 years' experience, almost 30% had >10 years' experience, and more than a quarter of the participants with >10 years' experience reported inadequate knowledge. Although, as acknowledged in the Limitations section, these numbers are reasonably small, which limits the generalisability of this study, the results indicate that this lack of knowledge may be persistent even in more experienced professionals. Further, as the genetics workforce is growing, with a large number of junior professionals, this data represents a proportion of the workforce whose training and knowledge needs must be addressed. The current HGSA guidelines on genetic counselling practice place responsibility on genetics professionals to discuss insurance issues with clients (11), but do not allocate responsibility for appropriate training and resourcing of professionals in this area. While the results suggest that that CGE's Fact Sheet 20 has been widely disseminated and most professionals are familiar with it, gaps in knowledge persist.

Almost all participants always discuss insurance implications of predictive testing with unaffected adult clients, despite evidence of professional knowledge limitations and a number self-reporting insufficient knowledge to adequately advise clients. This suggests that genetics professionals may not always provide correct information to clients on this issue. A Canadian study (9) has shown that genetic counsellors are comfortable discussing matters about which they are uncertain because discussions of uncertainty are routine in genetic counselling. In these circumstances, there is a risk that the legal implications could be poorly understood and incorrectly communicated to clients (9).

One mechanism to ensure consistent practice in Australasia is to include insurance implications on clinical consent forms signed by a client before genetic testing takes place, although this will not necessarily ensure informed consent. The findings showed variation across genetics services in this regard, indicating further inconsistency in client experience and mirroring international findings (17). The New South Wales Ministry of Health has recently implemented a new suite of consent forms for genetic and/or genomic testing in that state (18). It may be timely for genetics services to review their consent forms and ensure that the information on insurance issues are correct and consistent across sites. This will also assist with ensuring fully informed consent is obtained prior to testing, but would not negate the need for an explanation or discussion for many patients.

In addition, genetics services and state Health Departments, as well as interested bodies, such as the National Health and Medical Research Council (NHMRC) and the Australian Genomics Health Alliance (AGHA) could collaborate to build on existing precedents and develop nationally consistent training modules and resources, and model consent clauses that could be adapted to each clinic's needs. However, while a collaborative approach to the insurance implications of genetic testing will assist with national consistency, each genetics service must ultimately take final responsibility for maintaining appropriate policies, communicating them to staff and ensuring knowledge and practice are up-to-date. Given the potential financial implications of misinformation in this area for clients, action is needed to address this situation. Encouragingly, the findings of this study have already led to the implementation of some training initiatives in Victoria, and prompted the development of a patient brochure in conjunction with the Centre for Genetics Education.

A major finding of the study is the considerable professional concern regarding Australian regulation. Very few participants considered Australian regulations adequate, consistent with the HGSA's position statement regarding genetic testing and personal insurance products (10), which urges the implementation of a moratorium on the use of genetic test results.

There was no clear consensus among participants regarding what type of regulation should be implemented, though more participants agreed with the implementation of legislation than a moratorium (ban) on the use of genetic test results by insurers. It is argued elsewhere that a ban should be implemented (with the exception of mutation-negative results used to counter a family history of disease), along with longer term regulatory reform (19).

### Regulatory reform

The model of a self-regulated life insurance industry does not compel a rigorous standard of evidence regarding which genetic test results have a sufficient evidence base for use in underwriting (8). A key recommendation of the Australian Law Reform Commission's 2003 inquiry into the protection of genetic information (20) was the establishment of a body for this purpose. The Human Genetics Advisory Committee (HGAC) was established in November 2005 in response to this recommendation (21). Unfortunately, the HGAC has since been disbanded (22) and has not been replaced, meaning this recommendation has not been implemented. Any longer-term regulatory reform in this area should include a mechanism for oversight of the level of evidence that must be satisfied before genetic test results can be used for underwriting.

Regulatory reform on the use of genetic test results in life insurance underwriting must be considered by the Australian government to allow individuals to access genetic testing without fear of insurance implications. An inquiry into the life insurance industry has been conducted by the Joint Parliamentary Committee on Corporations and Financial Services. The Committee report, tabled in March 2018 (23), highlights the issues with industry self-regulation and the need for a moratorium on the use of genetic test results by insurers. At the time of writing, a moratorium has not been implemented. Unless the need for regulation is satisfactorily addressed, the potential for genetic testing to improve health outcomes for Australians will continue to be limited by insurance fears.

Our recommendations for addressing the issues identified in this paper are summarised in Table 2.

**Table 2:** Recommendations.

**No**	**Issue**	**Recommendation**
1	Some genetics professionals are inadequately equipped to advise client	Genetics services work with the HGSANHMRC, and the Centre for Genetics Education to develop training modules, resources and national guidelines regarding insurance issues, and maintain a regularly-updated resource page for access by genetics professionals.
2	Variability of professional practice	
3	Lack of consistency in consent forms	Genetics services work with the HGSA, state and territory Health Departments (with reference to the work already undertaken in NSW), the NHMRC and other interested bodies, such as the Australian Genomics Health Alliance (AGHA), to build on existing national precedents and develop national consent forms regarding genetic testing that include information about the insurance implications of genetic testing.
4	Regulation inadequate to protect clients from genetic discrimination	The Australian federal government must consider reforms regulating the use of genetic test results by insurers.

### Study limitations

The study has a number of limitations. Its relatively small sample size of 87 limits its generalisability, providing an indication of the issues that could be found in the broader professional workforce with further investigation. Participant demographics were skewed towards inexperienced professionals, with 59% having 0–10 years' experience and senior professionals (>20 years' experience), constituting only 8% of the sample. It is difficult to determine whether these percentages accurately represent the current workforce distribution, given the lack of publicly available data on this. All participants' experiences and attitudes were given equal weight, where participants with many years of experience and knowledge may have a more informed view. The survey questions could have better encompassed the New Zealand regulatory system to allow for more meaningful comparison.

The findings indicate an emerging clinical issue, highlighted by a lack of knowledge and/or training by a considerable proportion of genetics professionals. Individuals and health service organisations could better address the inconsistency of training provision and knowledge limitations in this area. Genetics services are responsible for developing appropriate policies and ensuring staff are adequately equipped in this regard. However, collaboration with other genetics services, the HGSA, and other relevant bodies, such as state governments, the National Health and Medical Research Council (NHMRC) and the Centre for Genetics Education (CGE), to develop a nationally consistent training programme, should be encouraged.

### Research recommendations

Future research could focus on exploring these issues in a larger cohort, as well as considering the content of genetic counselling sessions by direct transcript analysis, further investigation of the differences between clinical services in various Australasian locations, and consumer views and experiences regarding genetic testing and insurance issues.

## Author contributions

JT wrote the initial draft of the manuscript. PL substantially revised the manuscript. SW and LK provided research input to the development of the project and reviewed the manuscript and suggested revisions. MO and MD reviewed the manuscript and suggested revisions.

### Conflict of interest statement

The authors declare that the research was conducted in the absence of any commercial or financial relationships that could be construed as a potential conflict of interest.

## Footnotes

^1^Used here to refer to Australia and New Zealand.

^2^Health insurance is specifically protected from risk rating in Australia. This specific protection does not apply in New Zealand and so data from New Zealand participants was excluded from this question.

^3^It should be noted that FSC policies do not relate to travel insurance.
