# Supplementary material for: Genetics, Insurance and Professional Practice: Survey of the Australasian Clinical Genetics Workforce
Source: Front Public Health. 2018 Nov 23;6:333. doi: 10.3389/fpubh.2018.00333 (PMC6277853; doi:10.3389/fpubh.2018.00333)
Supplement: Supplementary file 1 [file Data_Sheet_1.PDF]

Murdoch Children's Research Institute

Insurance and Genetics

Codebook

Data Dictionary Codebook

08/02/2018 10:24

#	Variable / Field Name	Field Label<br>*Field Note*	Field Attributes (Field Type, Validation, Choices, Calculations, etc.)
Instrument: **Genetics and Insurance** (genetics_and_insurance)			
1	record_id	Record ID	text
2	plsread	Section Header: *Plain Language Statement and consent*<br>Please download and read the Plain Language Statement and indicate your willingness to proceed with the survey	descriptive
3	consent	By continuing with this survey I give my consent to being a participant in this research project	radio, Required<br>yes: Continue with survey<br>no: I do not want to continue with this survey<br>Stop actions on no
4	genprofyes	Do you work in a genetics service in which you have direct contact with clients who are considering genetic testing?	radio, Required<br>yes: Yes<br>no: No<br>Stop actions on no
5	genprofexplain<br>Show the field ONLY if:<br>[genprofyes] = 'no'	Section Header:<br>Thank you for your willingness to participate in the survey. Unfortunately, you do not meet the criteria for inclusion. Thanks anyway!	descriptive
6	sex	Section Header: *Demographic information*<br>Gender	radio, Required<br>1: Male<br>2: Female<br>3: Other/undisclosed
7	sexother<br>Show the field ONLY if:<br>[sex] = '3'		text
8	profqual	Profession	radio, Required<br>1: Clinical geneticist<br>2: Genetics fellow<br>3: Associate Genetic counsellor<br>4: Certified genetic counsellor<br>5: Other
9	profother<br>Show the field ONLY if:<br>[profqual] = '5'	Profession:	text, Required

10	pracyrsgc<br>Show the field ONLY if: [profqual] = '3' or [profqual] = '4'	How long have you been practising as a genetic counsellor?	radio, Required<br>1 0-5 years<br>2 6-10 years<br>3 11-15 years<br>4 15-20 years<br>5 more than 20 years
11	pracyrscg<br>Show the field ONLY if: [profqual] = '1'	How long have you been practising as a clinical geneticist?	radio, Required<br>1 0-5 years<br>2 6-10 years<br>3 11-15 years<br>4 15-20 years<br>5 more than 20 years
12	pracyrsgf<br>Show the field ONLY if: [profqual] = '2'	How long have you been practising as a genetics fellow?	radio, Required<br>1 0-5 years<br>2 6-10 years<br>3 11-15 years<br>4 15-20 years<br>5 more than 20 years
13	apptcount	On average, how many appointments by phone or in person do you take per fortnight?	radio, Required<br>1 0-5<br>2 6-10<br>3 11-20<br>4 more than 20
14	workstate	Where is the genetics service where you primarily work located?	radio, Required<br>1 ACT<br>2 NSW<br>3 NT<br>4 NZ<br>5 QLD<br>6 TAS<br>7 VIC<br>8 WA<br>9 SA
15	primrururb	Your primary workplace is	radio, Required<br>1 Urban<br>2 Rural/regional<br>3 Both
16	sector	In what sector is the genetics service where you primarily work?	radio, Required<br>1 Private sector<br>2 Public sector<br>3 Both<br>4 Other
17	sectorother<br>Show the field ONLY if: [sector] = '4'	Other:	text

18	training	Section Header: *Training and education (current workplace)*<br>Has your genetics service provided any training regarding insurance implications of genetic testing? [select all that apply]	checkbox, Required<br>Formal | training___formal | Yes, formal training<br>Informal | training___informal | Yes, informal training<br>no | training___no | No
19	trainsuff<br>Show the field ONLY if: [training(Formal)] = '1' or [training(Informal)] = '1'	Do you feel this training has been adequate?	radio, Required<br>yes | Yes<br>no | No
20	trainsuffelab	Please elaborate<br>*Optional field*	notes
21	trainneed<br>Show the field ONLY if: [training(no)] = '1'	Do you consider you need training in this area?	radio, Required<br>yes | Yes<br>no | No
22	knowconf	Do you feel you have sufficient knowledge about the insurance implications of genetic testing to properly advise clients?	radio, Required<br>yes | Yes<br>no | No<br>unsure | I don't know
23	knowconfexplain<br>Show the field ONLY if: [knowconf] = '3' or [know conf] = '4'	What, if anything, would make you feel more confident?<br>*Optional field*	notes
24	inscrit	How do you determine whether to raise insurance implications with each client?	radio, Required<br>1 | I use set written criteria<br>2 | I use set criteria that is not written down<br>3 | Based on my personal experience and knowledge<br>4 | I don't know
25	inscritexplain	Please elaborate<br>*Optional field*	notes
26	unsurediscuss	Are you ever unsure about when to discuss insurance implications with clients?	radio, Required<br>yes | Yes<br>no | No
27	agreedpol	Does your genetics service have an agreed policy regarding communicating with clients about insurance implications of genetic testing?	radio, Required<br>written | Yes, a written policy<br>verbal | Yes - a verbal policy that has been discussed with me or at meetings at which I was present<br>no | No<br>unsure | I don't know
28	poladeq<br>Show the field ONLY if: [agreedpol] = 'written' or [agreedpol] = 'verbal'	Do you consider the policy is adequate?	radio, Required<br>yes | Yes<br>no | No<br>unsure | I don't know
29	polneed<br>Show the field ONLY if: [agreedpol] = 'no' or [agr eedpol] = 'unsure'	Do you think your genetics service should have a policy?	radio, Required<br>yes | Yes<br>no | No<br>unsure | I don't know

30	polneedelab	Please elaborate<br>*Optional field*	notes
31	readhgsa	Section Header: *Has your genetics service encouraged you to be familiar with, and have you read, the following fact sheets regarding insurance and genetics? You can access and view copies of these documents below if you wish.*<br>The HGSA position statement on Genetic Testing and Life Insurance	radio (Matrix), Required<br>yes: Yes, and I have read it<br>notread: Yes, but I have not read it<br>haveread: No, but I have read it<br>no: No
32	readcge	Fact Sheet 20 published by the Centre for Genetics Education	radio (Matrix), Required<br>yes: Yes, and I have read it<br>notread: Yes, but I have not read it<br>haveread: No, but I have read it<br>no: No
33	readfsc11	The Financial Services Council (FSC) Policy No 11 on Genetic testing	radio (Matrix), Required<br>yes: Yes, and I have read it<br>notread: Yes, but I have not read it<br>haveread: No, but I have read it<br>no: No
34	readfscfact	The Investment and Financial Services Association (IFSA - now FSC) fact sheet on life insurance and genetic testing in Australia	radio (Matrix), Required<br>yes: Yes, and I have read it<br>notread: Yes, but I have not read it<br>haveread: No, but I have read it<br>no: No
35	readnhmrc	The NHMRC guidelines relating to medical genetic testing	radio (Matrix), Required<br>yes: Yes, and I have read it<br>notread: Yes, but I have not read it<br>haveread: No, but I have read it<br>no: No
36	viewhgsa		descriptive
37	viewfs20cge		descriptive
38	viewfsc11		descriptive
39	readifsa		descriptive
40	viewnhmrc		descriptive
41	factoth	Are you aware of any other organisations' fact sheets or information documents that are relevant to genetic testing and insurance?<br>*Optional field*	notes
42	consentformtype	Section Header: *Practice*<br>In your genetics service, when obtaining your clients' consent for genetic testing	radio, Required<br>1: There is one standard consent form used for all testing<br>2: There are several different consent forms
43	standardconforminsurance<br>Show the field ONLY if: [consentformtype] = '1'	Does the standard consent form include a statement about insurance implications?	radio, Required<br>yes: Yes<br>no: No

44	differentconforminsurance<br>Show the field ONLY if: [consentformtype] = '2'	The genetic testing consent forms	radio, Required<br>1 All contain statements about insurance implications<br>2 Some contain statements about insurance implications<br>3 None contain statements about insurance implications
45	consentinsuranceexplain<br>Show the field ONLY if: [differentconforminsurance] = '2'	Please elaborate<br>*Optional field*	notes
46	predadultform<br>Show the field ONLY if: [consentformtype] = '2'	Do you have a specific form for predictive testing in adults?	radio, Required<br>yes Yes<br>no No
47	predadultformins<br>Show the field ONLY if: [predadultform] = 'yes'	Does the form contain a statement about insurance implications?	radio, Required<br>yes Yes<br>no No
48	diagchn	Section Header: *Do you discuss insurance implications with clients in the following contexts? If you do not see clients in these contexts, please tick "N/A"*<br>Diagnostic testing in children	radio (Matrix), Required<br>1 Always<br>2 Sometimes<br>3 Rarely<br>4 Never<br>5 N/A
49	diagadult	Diagnostic testing in adults	radio (Matrix), Required<br>1 Always<br>2 Sometimes<br>3 Rarely<br>4 Never<br>5 N/A
50	predchn	Predictive testing in unaffected children	radio (Matrix), Required<br>1 Always<br>2 Sometimes<br>3 Rarely<br>4 Never<br>5 N/A
51	predadult	Predictive testing in unaffected adults	radio (Matrix), Required<br>1 Always<br>2 Sometimes<br>3 Rarely<br>4 Never<br>5 N/A
52	pntaneup	Prenatal testing	radio (Matrix), Required<br>1 Always<br>2 Sometimes<br>3 Rarely<br>4 Never<br>5 N/A

53	secfinding	The return of secondary findings	radio (Matrix), Required<br>1 Always<br>2 Sometimes<br>3 Rarely<br>4 Never<br>5 N/A
54	somecircatt	Please elaborate<br>*Optional field*	notes
55	insimpoth	Are there any other circumstances in which you consider insurance implications are relevant?<br>*Optional field*	notes
56	inscommimp	Section Header: *The following questions relate to predictive genetic testing of adults, based on your experience over the past 12 months*<br>Communicating information about insurance implications of predictive genetic testing in adults is:	radio, Required<br>1 Very important<br>2 Somewhat important<br>3 Not particularly important<br>4 Not important at all
57	inscommresp	Communicating the implications of genetic testing with your clients is [select all that apply]:	checkbox, Required<br>your — inscommresp___your<br>workother — inscommresp___workother<br>outworkother — inscommresp___outworkother<br>nobody — inscommresp___nobody<br>Unsure — inscommresp___unsure
58	responsibexplain<br>Show the field ONLY if: [inscommresp(workother)] = '1' or [inscommresp(outworkother)] = '1' or [inscommresp(nobody)] = '1'	Please elaborate<br>*Optional field*	notes
59	insdisccouns	Section Header: *Discussion about insurance implications is initiated by*<br>You	radio (Matrix), Required<br>1 Most of the time<br>2 Some of the time<br>3 Rarely
60	insdiscclnt	Your client	radio (Matrix), Required<br>1 Most of the time<br>2 Some of the time<br>3 Rarely

61	instypesuse	For which types of insurance policies can genetic information be used in Australia? [select all that apply]	checkbox, Required<br>health | instypesuse___health | Private health insurance<br>life | instypesuse___life | Life insurance<br>inc | instypesuse___inc | Income protection insurance<br>dis | instypesuse___dis | Disability insurance<br>trv | instypesuse___trv | Travel insurance<br>unsure | instypesuse___unsure | I don't know
62	inspolaff	Which types of insurance policies can be affected by genetic test results?	radio, Required<br>new | New policies applied for after genetic testing<br>existing | Existing policies that were taken out before genetic testing<br>both | Both types of policies<br>unsure | I don't know
63	inspolaffelab	Please elaborate<br>*Optional field*	notes
64	onlyrel	Section Header: *The reasons why you might NOT discuss insurance implications for adult predictive testing are:*<br>In some circumstances it is not relevant for a client	radio (Matrix), Required<br>1 | Agree<br>2 | Disagree
65	servnotimp	It is not a priority for your genetics service	radio (Matrix), Required<br>1 | Agree<br>2 | Disagree
66	forget	Sometimes you forget	radio (Matrix), Required<br>1 | Agree<br>2 | Disagree
67	notime	You do not have enough time	radio (Matrix), Required<br>1 | Agree<br>2 | Disagree
68	deter	You are concerned that it might deter clients from having genetic testing	radio (Matrix), Required<br>1 | Agree<br>2 | Disagree
69	insdiscoth	Are there other reasons why you might not discuss insurance implications of adult predictive testing with clients?<br>*Optional field*	notes
70	insimpcontentself	As a summary, what, if anything, do you tell clients about the insurance implications for THEMSELVES of adult predictive genetic testing?<br>*Optional field*	notes
71	insimpcontentfam	As a summary, what, if anything, do you tell clients about the insurance implications for their FAMILY MEMBERS of adult predictive genetic testing?<br>*Optional field*	notes

72	orgins	How often do you discuss with clients the option to go away and organise their insurance before having genetic testing?	radio, Required<br>1 always<br>2 often<br>3 sometimes<br>4 never
73	tanfraud	There has been some suggestion by insurance companies that this may amount to fraud. Do you agree?	radio, Required<br>yes Yes<br>no No<br>unsure I don't know
74	tanfraudelab	Please elaborate<br>*Optional field*	notes
75	insimpclireact	What is the most common reaction clients have to information about insurance implications of genetic testing?	radio, Required<br>1 anxiety<br>2 confusion<br>3 fear<br>4 nonchalance<br>5 lack of concern<br>6 other
76	clireactotherlab<br>Show the field ONLY if:<br>[insimpclireact] = '6'		text
77	pracchange	Have you changed your practice in terms of what information you give to clients about insurance implications of genetic testing over the past 12 months?	radio, Required<br>yes Yes<br>no No
78	pracchangeexplain<br>Show the field ONLY if:<br>[pracchange] = 'yes'	Please elaborate<br>*Optional field*	notes
79	insuseconcern	Do you feel that the more information you provide about insurance implications, the more it deters clients from having genetic testing?	radio, Required<br>yes Yes<br>no No<br>unsure I don't know
80	insimpclielab	Please elaborate<br>*Optional field*	notes
81	notproceedlife	How often do you estimate clients that you have seen refuse genetic testing because of life, income or disability insurance concerns?	radio, Required<br>1 often<br>2 sometimes<br>3 rarely<br>4 never
82	detailnotproceedlife	Further details (if applicable):	notes
83	notproceetrv	How often do you estimate clients that you have seen refuse genetic testing because of travel insurance concerns?	radio, Required<br>1 often<br>2 sometimes<br>3 rarely<br>4 never
84	detailnotproceedtrv	Further details (if applicable):	notes

85	inscompcontact	Have insurance companies contacted you directly about genetic test results of your client/s?	radio, Required<br>yes Yes<br>no No
86	inscontacteg<br>Show the field ONLY if: [inscompcontact] = 'yes'	Please provide examples<br>*Optional field*	notes
87	inscospeak	Have you spoken with insurance companies regarding decisions they have made about your clients on the basis of genetic test results?	radio, Required<br>yes Yes<br>no No
88	insspeakeg<br>Show the field ONLY if: [inscospeak] = 'yes'	Please provide examples<br>*Optional field*	notes
89	gentestadv	Have you had direct experience with a client/s who has had an adverse policy outcome on the basis of genetic test results?	radio, Required<br>yes Yes<br>no No
90	gentestadvelab	Please elaborate<br>*Optional field*	notes
91	considagree	Section Header: *Opinions*<br>The current version of the Financial Services Council Genetic Testing Policy recommends that life insurers ask applicants:<br><br>"Have you ever had or are you considering having a genetic test where you have received (or are currently awaiting) an individual result?"<br><br>Do you think that this should be allowed?	radio, Required<br>yes Yes<br>no No<br>unsure I don't know
92	consagreeelab	Please elaborate<br>*Optional field*	notes
93	considerchangadv	Would this change the way you advise clients?	radio, Required<br>yes Yes<br>no No
94	discdeterelab	Please elaborate<br>*Optional field*	notes
95	regadeq	Do you think the current regulations in Australia regarding insurance and genetics are adequate to protect clients from genetic discrimination?	radio, Required<br>yes Yes<br>no No<br>unsure I don't know
96	regelab	Please elaborate<br>*Optional field*	notes
97	morator	Do you think Australia should have a moratorium (ban) on the use of genetic information by life, disability, income protection and travel insurance companies?	radio, Required<br>yes Yes<br>no No<br>unsure I don't know
98	moraelab	Please elaborate<br>*Optional field*	notes
99	legis	Do you think Australia should have separate legislation regulating the use of genetic information by life, disability, income protection and travel insurance companies?	radio, Required<br>yes Yes<br>no No<br>unsure I don't know

100	legelab	Please elaborate<br>*Optional field*	notes
101	commentoth	Section Header: *Final comments and follow-up*<br>Do you have any other comments to add about any of the matters raised in this survey?<br>*Optional field*	notes
102	recontactconsent	Section Header: *Further contact*<br>As part of this research project, we may want to contact you to discuss the matters raised in this survey further. Any data collected in this follow-up interview will be de-identified before being published or shared.<br><br>If you consent to being contacted for a follow-up interview, please provide your contact details below. Please note that you are free to remain anonymous and decline to provide your contact details to us if you prefer.	radio, Required<br>anonymous: I prefer to remain anonymous<br>contactme: I am happy to be contacted in the future
103	contactname<br>Show the field ONLY if: [recontactconsent] = 'contactme'	Name	text, Required, Identifier
104	contactemail<br>Show the field ONLY if: [recontactconsent] = 'contactme'	Email address	text (email), Required, Identifier
105	contactphone<br>Show the field ONLY if: [recontactconsent] = 'contactme'	Best telephone contact number	text, Required, Identifier
106	genetics_and_insurance_complete	Section Header: *Form Status*<br>Complete?	dropdown<br>0: Incomplete<br>1: Unverified<br>2: Complete
